# Case Report: Wernicke's encephalopathy after gastric surgery presenting as lactic acidosis and refractory thrombocytopenia

**DOI:** 10.3389/fsurg.2023.1016347

**Published:** 2023-02-21

**Authors:** Qi Lin, Guanghua Li, Zhixiong Wang, Yu Zhang

**Affiliations:** ^1^ Department of Gastrointestinal Surgery, The First Affiliated Hospital of Sun Yat-sen University, Guangzhou, China; ^2^ Department of Neurology, The First Affiliated Hospital of Jinan University, Guangzhou, China

**Keywords:** Wernicke's encephalopathy, lactate, thrombocytopenias, gastric cancer, postoperative management

## Abstract

Wernicke's encephalopathy (WE) is a severe neuropsychiatric disorder, mainly resulting from a nutritional deficiency of thiamine. WE is hard to detect at an early stage. Less than 20% of WE can be diagnosed during a patient’s lifetime, and WE tends to occur in patients with chronic alcoholism. Therefore, a large proportion of non-alcoholic WE patients are misdiagnosed. Lactate is an important by-product of anaerobic metabolism when the aerobic metabolism is blocked without thiamine, which can potentially serve as an alerting index for WE. Here, we report a case of a patient with WE who suffered gastric outlet obstruction following postoperative fasting, accompanied by lactic acidosis and refractory thrombocytopenia. A 67-year-old non-alcoholic woman who suffered hyperemesis for 2 months was diagnosed with gastric outlet obstruction (GOO). Gastric biopsies with endoscopy revealed gastric cancer, and total gastrectomy, together with D2 nodal dissection, was performed. She developed a coma with refractory thrombocytopenia rapidly after the surgical procedures were performed. The above conditions were treated not by the administration of antibiotics but by that of thiamine. We also found before the start of the procedures that she had a high level of blood lactate for a long period of time. Early diagnosis of WE is important because permanent injury can be caused to the central nervous system. Even today, the diagnosis of WE mainly depends on clinical symptoms, but occasionally, a typical triad occurs among WE patients. Therefore, a sensitive index for early diagnosis is critical for WE. Rising levels of blood lactate as a result of thiamine deficiency can serve as a warning for WE. In addition, we noted that this patient had a non-typical thiamine-sensitive refractory thrombocytopenia.

## Introduction

Wernicke's encephalopathy (WE) is a series of neurological disorders caused by a deficiency of thiamine, and its main symptoms are ophthalmoplegia, ataxia, and mental confusion, together defined as a “typical triad.” Thiamine, also known as Vitamin B1, plays a vital role in the cardiovascular, muscular, nervous, and gastrointestinal systems in the human body. As a water-soluble vitamin, thiamine is stored only limitedly in liver, and it will run out in 18–20 days without adequate supplements (1). Wernicke's encephalopathy is hard to detect at an early stage. Less than 20% of WE can be diagnosed during a patient’s lifetime, and WE tends to occur in patients with chronic alcoholism (2). Therefore, a large proportion of non-alcoholic WE patients are misdiagnosed. Patients who undergo a gastrointestinal surgery, who suffer from the conditions of chronic malnutrition, prolonged parenteral nutrition, and hyperemesis, or rarely malignant tumor, can be susceptible to WE (3, 4). Testing the thiamine levels in plasma is technically feasible, but it is constrained by its poor sensitivity and specificity (5).

Thiamine is an essential co-factor for pyruvate dehydrogenase in aerobic glucose metabolism. The deficiency of thiamine will cause a retardation of aerobic metabolism, and anaerobic metabolism will be activated instead. Consequently, the levels of lactate, one of the products of anaerobic metabolism, will rise steeply. Changes in blood lactate levels can be an important alert for WE.

Here, we report a case of a patient diagnosed with gastric cancer, who developed WE after gastrectomy because of insufficient vitamin supplements and presented with a confusional state, persistent high levels of lactate, and thrombocytopenia.

## Case report

A 67-year-old non-alcoholic female complained of progressive vomiting for 2 months, following which she lost more than 2.5 kg of weight for 1 month before being admitted to the gastrointestinal department. An abdominal computerized tomography (CT) screening indicated gastric outlet obstruction. A biopsy from gastric endoscopy confirmed low-differentiated gastric adenocarcinoma. Subsequently, she consented for a radical total gastrectomy, D2 lymph node dissection, and esophagojejunostomy. We initiated her fasting, venous antibiotics, and parenteral nutrition after the surgical procedures.

**Figure 1:**
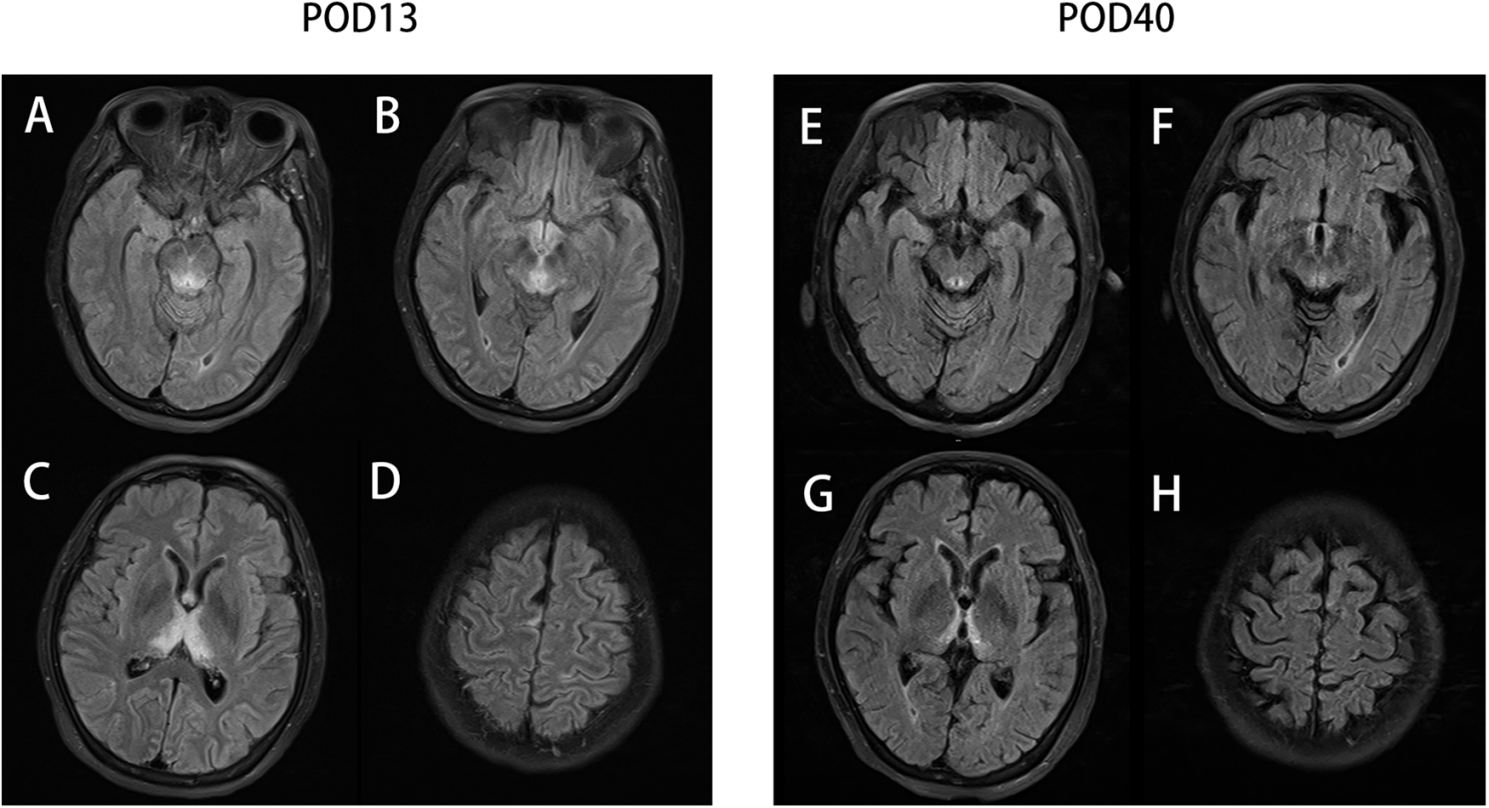
The T2-Flair sequencing MR image of the periaqueductal region of the midbrain (**A**), mammillary bodies (**B**), thalamus (**C**), and parietal cortices (**D**) on postoperative day 13 (POD13) and the T2-Flair sequencing MR image of the periaqueductal region of the midbrain (**E**), mammillary bodies (**F**), thalamus (**G**), and parietal cortices (**H**) on POD 40.

On postoperative day (POD) 1, the serum procalcitonin (PCT) of this patient rose rapidly from 0.12 μg/L (before surgery) to 5.46 μg/L, along with an increase in body temperature. These signs prompted a diagnosis of postoperative infection, which was the main misleading hypothesis in our following clinical decisions; thus, we substituted cefoperazone-sulbactam for cefuroxime to enhance antibiosis. On POD 3, the patient felt dizziness and nausea, with her forehead sweating. Low blood glucose levels (2.9 mmol/L) confirmed our diagnosis of hypoglycemia, so we cut down the usage of the venous total nutrient admixture (containing insulin) and simultaneously gave her a venous glucose infusion. On the night of POD 4, sudden delirium and hypersomnia manifested in this patient, along with a dull pupillary light reflex. The blood C-reactive protein (CRP) level was high (226.57 mg/L), along with a rising temperature, quickening heart/respiratory rate, and low white blood cell (WBC) count (3.08*10^9^/L), indicating worsening infection or even systemic inflammatory response syndrome (SIRS). For the purpose of locating the focal infection, we performed an emergency CT, and we ruled out anastomotic stoma leakage, surgical site infection (SSI), or severe abdominal infection because of no relevant signs in abdominal CT screening and her soft abdominal muscle. But the chest CT detected bilateral pleural effusion and atelectasis, so we estimated them as pulmonary infection and hyperdynamic infectious shock because of her temporary hemodynamic stability. Accordingly, we upgraded the antibiotics (vancomycin + meropenem). However, such finer antibiotics did not stop her consciousness from deteriorating. On POD 7, the patient lay in a state of deep coma, with the absence of any response to stitching and pupillary light reflex. We initiated a consultation with the neurology department to deal with the patient's poor neurological condition. A colleague in the department disagreed with the view that the neurological changes might be correlative to endocranial lesion instead of infection. To ascertain the reason for her state of unconsciousness, we performed a craniocerebral magnetic resonance imaging (MRI) on this patient, which revealed abnormal signals from the bilateral medial thalamus, third ventricle, posterior brainstem, and periaqueductal gray matter (Figure 1A). Considering these results and the opinion obtained from the neurological department, a diagnosis of Wernicke's encephalopathy was made.

**Figure 2:**
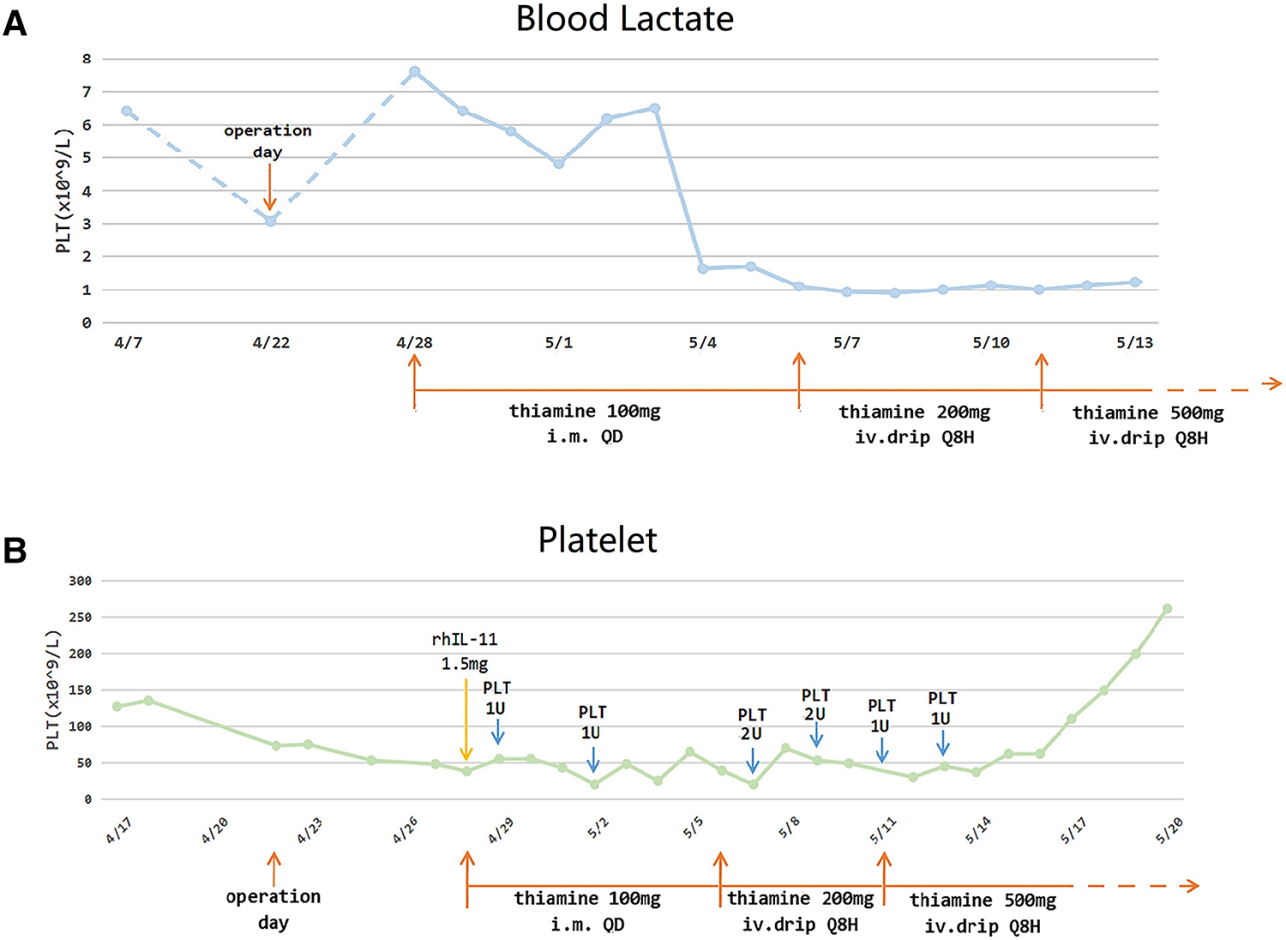
The variations in the blood lactate level (**A**) and platelet count (**B**).

It should be noted that the venous supplement of thiamine (100 mg per day) had been initiated in accordance with the suggestion from the nutritional department since POD 6. We subsequently increased the dosage of thiamine (200 mg every 8 h) as a remedy for WE. As expected, the level of consciousness of this patient soon increased (mildly responsive to stitching and an insensitive pupillary light reflex). The supplement of thiamine was reinforced (500 mg every 8 h) 3 days later (Figure 2A). Even the pupillary light reflex and the stitching response improved, which contrasted with the situation that obtained on POD 7; however, the patient was still in a state of unconsciousness and was transferred to the ICU for further treatment. A craniocerebral MRI on POD 40 (Figure 1B) indicated a shrunken lesion in her brain, providing another reliable evidence on the effectiveness of thiamine therapy. But the MRI also revealed disappear irreversible destruction of periaqueductal neurocytes, which was an alert for poor prognosis. Long-term confinement in the bed led to hypostatic pneumonia and septic shock. Eventually, the patient died on POD 245 because of secondary respiratory failure.

There are two noteworthy points in this case: (1) We found that 15 days before this patient consented for the surgical procedures, her blood lactate level (6.4 mmol/L) had highly surpassed normal levels (0.5–1.7 mmol/L). We inferred that the vomiting that she experienced for 2 months had resulted in thiamine deficiency, and long-term alternative anaerobic metabolism led to the accumulation of lactate. Correspondingly, her blood lactate levels began to decline gradually when we enhanced thiamine supplementation (Figure 2A) and found her response to stitching improve. (2) We noticed that her platelet (PLT) count decreased acutely since POD 1 (53 × 10^9^/L vs. 135 × 10^9^/L before surgery), and this index kept dropping to 38 × 10^9^/L on POD4, in spite of the multiple applications of rhIL-11 and platelet transfusion. But the refractory thrombocytopenia started healing after enhanced thiamine supplementation, and simultaneously, the blood lactate levels dropped and consciousness improved. Eventually, the blood PLT count increased to 149 × 10^9^/L and remained stable (Figure 2B).

## Discussion

This is a case report of a non-alcoholic patient who developed Wernicke's encephalopathy after gastrointestinal surgery. Thiamine, also known as Vitamin B1, is an important element of the pyruvate dehydrogenase complex (PDHC) and α-ketoglutarate dehydrogenase complex (ADHC). A deficiency of these complexes reduces the efficiency of the tricarboxylic acid cycle. When this occurs in the central nervous system, subsequent lactic accumulation and ATP-dependent Na^+^–K^+^ pump devitalization will cause neurocyte edema and further cellar apoptosis; this is the main mechanism of Wernicke's encephalopathy (6, 7). As a water-soluble vitamin, 1–2 mg thiamine is excreted in the urine and gets discharged without ample replenishment (5). The European Federation of Neurological Societies (EFNS) suggests a three-time 200 mg thiamine intravenous supplement for non-alcoholic WE patients, and thiamine therapy has proved extremely safe for most patients (8–10).

Wernicke's encephalopathy can occur in postoperative patients in the surgical ward because of the presence of risk factors such as diarrhea, malnutrition, and so on. Particularly in the gastrointestinal surgical ward, postoperative fasting is necessary for most patients, which gives rise to a risk for vitamin deficiency. What is worse, postoperative glucose infusion for the purpose of enhancing nutrition also leads to thiamine deficiency because glucose metabolism exhausts thiamine as well (11). Approximately 20%–30% of gastric cancer patients present with a stage IV disease. Similar to our present case, antropyloric advanced cancer frequently leads to gastric outlet obstruction (GOO). A GOO patient tends to present with vomiting after food intake, and such intake disorder can cause severe malnutrition and fluid and electrolyte imbalances that are difficult to control (12, 13). The prognosis for WE patients depends on whether thiamine therapy is initiated at an early stage. Such patients usually respond well to timely treatment, recovering from their conditions of altered mental status and acute encephalopathy (apathy, drowsiness, confusion, etc.), while residual neurologic function deficits (memory, learning deficits, etc.) tend to persist (10). Unfortunately, most patients miss the optimum treatment because of misdiagnosis, resulting in irreversible symptoms, permanent Korsakov amnesia, and an almost 20% mortality rate (14). Therefore, early diagnosis and treatment of Wernicke encephalopathy are extremely important factors impacting prognosis.

The diagnosis of WE depends on the presence of the clinical typical triad (ophthalmoplegia, ataxia, and mental confusion). The diagnosis sensitivity of the typical triad is 23%, but it rises to 85% with at least two of the four following features: dietary deficiencies, eye signs, cerebellar signs, and either memory impairment or an altered mental state (8, 15). However, these clinical features are less relevant for patients without any alcoholic history, because most of them show no other symptom except an altered mental state. The eye signs of WE usually present as nystagmus, which was absent in our patient, but she manifested a dull pupillary light reflex instead, which was atypical (16). Cerebellar signs are usually not distinct in a bedridden postoperative patient.

The pathophysiological changes in brain cytotoxic edema and vasogenic edema are caused by a dysfunction of the Krebs cycle and the pentose phosphate pathway (17). Typically, the lesions are symmetrical and seen in the thalami, mamillary bodies, corpora quadrigemina, and periaqueductal area. Atypical lesions could be located in the cerebellum, vermis, cranial nerve nuclei, red nuclei, dentate nuclei, caudate nuclei, splenium, and cerebral cortex (5). MRI is a sensitive examination for discovering the cerebral lesion of WE. A total of 99% lesions can be revealed by conventional MRI and 100% by fluid-attenuated inversion recovery (FLAIR) sequence MRI in non-alcoholic patients, and nearly two-third alcoholic cases can be detected by FLAIR-MRI. MRI is also sensitive in evaluating disease progression (8). But with MRI being an expensive examination, it is rarely performed before patients slip into coma or other severe mental states, which limits its utilization in early diagnosis.

Laboratory tests can be helpful. Today, it is possible to measure blood thiamine or thiamine pyrophosphate (TPP) concentration directly, which is the gold standard for diagnosing thiamine deficiency. But it is not an examination with satisfactory levels of sensitivity, and the parameter of normal thiamine levels is not a criterion to exclude WE from diagnosis because of the decreasing levels of inflammation-related thiamine (3, 18). Notably, the testing of blood thiamine levels is untrustworthy and relatively time-consuming, so the practice of waiting for the laboratory result to start thiamine supplement is not strongly encouraged or recommended (8).

Elevated lactate levels are often used as a prognostic tool in critical illness. There are many causes for lactic acidosis, including sepsis, systemic hypoperfusion, medications, and thiamine deficiency (9). Lactic acid accumulates as a by-product of anaerobic metabolism when thiamine is not sufficient to actuate aerobic metabolism. Therefore, rising blood lactate levels showing up with the relevant risk factors should be an alert for potential thiamine deficiency. Although a change in lactate concentration is not a specific indicator for diagnosing thiamine deficiency, it is still a guiding factor for performing an MRI examination and providing preventive thiamine treatment. In our patient, lactate concentration increased steeply after she suffered gastric outlet obstruction, but it returned to normal rapidly after the supplement of thiamine, which proves that changes in lactate levels are correlative to the actual physical condition arising out of thiamine supplementation.

What should be noted here is that, this patient presented with a persistent and poorly responsive thrombocytopenia after surgery, but the thrombocytopenia healed subsequently with thiamine supplementation. Thrombocytopenia is not a common feature of thiamine deficiency. A similar refractory thrombocytopenia (34*10^9^/L) of a WE patient was reported by Stefano et al. They attributed the patient’s septicopyemia-related myelosuppression to thrombocytopenia, because it faded with other infective signs (19). But in our patient, thrombocytopenia levels kept decreasing regardless of antibiotic upgrading, and this situation was reversed only by giving thiamine supplement, which indicated the correlation between thiamine deficiency and thrombocytopenia. The case of another patient with WE analogously revealed a thiamine-responsive thrombocytopenia, reported by Francesco et al. (20). However, the mechanism between thiamine and thrombocytopenia remains to be explored. Notably, thrombocytopenia is a typical feature of thiamine-responsive megaloblastic anemia (TRMA) syndrome. TRMA is an autosomal recessive disorder resulting from disturbed cellar thiamine uptake. A tetralogy of TRMA includes megaloblastic anemia, mild thrombocytopenia and leukopenia, sensorineural deafness, and diabetes mellitus (21). Treatment with pharmacologic doses of thiamine cures the megaloblastic anemia and diabetes mellitus in both TRMA and dietary thiamine-deficient patients. But it still unclear why symptoms differ in these patients (21). The megaloblastic anemia of TRMA is mainly attributed to RNA ribose synthesis deficiency, relative to the non-oxidative transketolase pathway (22). The reason for the blocking of this pathway in TRMA cells is the mutation of high-affinity components of the thiamine transporter on the cytomembrane, SLC19A2 (21, 23). Thus, even a mild thiamine deficiency can cause obvious megaloblastic anemia in a TRMA patient, because the deficiency is ineffective to induce the cerebral lesion. Oppositely, encephalopathy shows up early in a dietary thiamine-deficient patient. We propose that cellar thiamine deficiency is more likely to cause thrombocytopenia. In our patient, thiamine deficiency was so severe that not only the brain but also her primitive blood cells were affected. Even though SLC19A2 functioned normally, thrombocytopenia occurred because of cellar thiamine deficiency.

Here, we report a case of a patient with postoperative Wernicke’s encephalopathy to highlight the importance of early diagnosis and treatment for WE patients. A high blood lactate concentration can trigger an alarm for WE, although the importance of blood lactate for early diagnosis needs further exploration. On the other hand, we also reported the condition of thiamine-responsive thrombocytopenia in this patient, which is refractory even with extensive blood platelet transfusion and the availability of thrombopoietin.

## Learning points

1. A postoperative fasting patient is susceptible to Wernicke’s encephalopathy. Because WE tends to be indiscoverable and irreversible, preventative water-soluble vitamin administration should be implemented after a gastrointestinal surgery.
2. Lactic acidosis is not specific for the diagnosis of WE, but we consider that it is worth performing a cerebral magnetic resonance screening on a fasting patient with lactic acidosis, even though no typical neurological signs are visible.
3. This study reports a rare thiamine-sensitive thrombocytopenia in WE, and its mechanism has yet to be studied and verified.

## Data Availability

The original contributions presented in the study are included in the article/**Supplementary Material;** further inquiries can be directed to the corresponding author.
